# Origin and speciation of *Picea schrenkiana* and *Piceasmithiana* in the Center Asian Highlands and Himalayas

**DOI:** 10.1007/s11105-014-0774-5

**Published:** 2014-08-17

**Authors:** Lili Li, Yongshuai Sun, Jiabin Zou, Wei Yue, Xi Wang, Jianquan Liu

**Affiliations:** State Key Laboratory of Grassland Agro-Ecosystems, School of Life Sciences, Lanzhou University, Lanzhou, 730000 China

**Keywords:** Gene flow, *Picea schrenkiana*, *P. smithiana*, Population genetic data, Speciation test

## Abstract

Elucidating the evolutionary history of current species diversity, especially trees with large effective population sizes and long generation times, is a complicated exercise confounded by gene flow and incomplete lineage sorting. In the present study, we aim to determine the origin and speciation of *Picea schrenkiana* and *Picea smithiana* using population genetic data from chloroplast (cp), mitochondrial (mt), and nuclear (nr) genomes. These two species occur in the Central Asian Highlands and Himalayas, respectively, where they are isolated from other Asian congeneric species by the Qinghai–Tibet Plateau (QTP) or adjacent deserts. Previous studies based on both morphological and molecular evidence suggest that they have contrasting phylogenetic relationships with *Picea likiangensis* or *Picea wilsonii* which are closely related and both located in the QTP. We examined genetic variation among 16 loci of three genomes from 30 populations of these four species. At both cpDNA loci and mtDNA loci, *P. schrenkiana* appeared to be closely related to *P. likiangensis*, although statistical support for this was weak. However, phylogenetic analyses and speciation tests based on the nuclear data from 11 loci provided evidence that *P. schrenkiana* and *P. smithiana* are sister species. These two species diverged around five million years ago (Mya) while the divergence between them and the *P. likiangensis*–*P. wilsonii* clade occurred about 18.4 Mya. We also detected gene flow accompanying these speciation events. Our results highlight the complex speciation histories of these alpine conifers due to interspecific gene flow and/or incomplete lineage sorting, and the importance of the early QTP uplifts in promoting the origin of these important conifer species in the Asian highlands.

**Electronic supplementary material:**

The online version of this article (doi:10.1007/s11105-014-0774-5) contains supplementary material, which is available to authorized users.

## Introduction

Geographical or other selective barriers to genetic exchange may give rise to new species by isolating previously interbreeding populations (Coyne and Orr 2004). Such geographical isolation can play an important role in dividing a common ancestor into two (rarely more) sister species in which different random alleles become fixed and adaptions to different local conditions are selected (Mayr 1954; Grant 1981; Coyne 1992; Orr and Presgraves 2000). In addition, gene flow and incomplete lineage sorting complicate speciation process, especially in tree species with large effective population sizes and long generation times (Gavrilets 2003; Levin 2003; Rieseberg and Willis 2007). Evidence increasingly suggests that population-genetic data, derived from sequencing multiple loci, is the best approach for clarifying the origin and speciation of such tree species (e.g., Chen et al. 2010; Zou et al. 2013; Sun et al. 2014). Such data allow for alternative speciation models and estimation of the extent of gene flow that has accompanied speciation (e.g., Hey and Nielsen 2004, 2007; Hey 2006, 2010a, b; Li et al. 2010). In addition, estimates of divergence timescales based on analysis of multilocus population genetic data in a coalescent framework provide good temporal hierarchies for understanding the roles of geological events in triggering speciation (Wakeley 2003; Takayama et al. 2013).

**Fig. 1:**
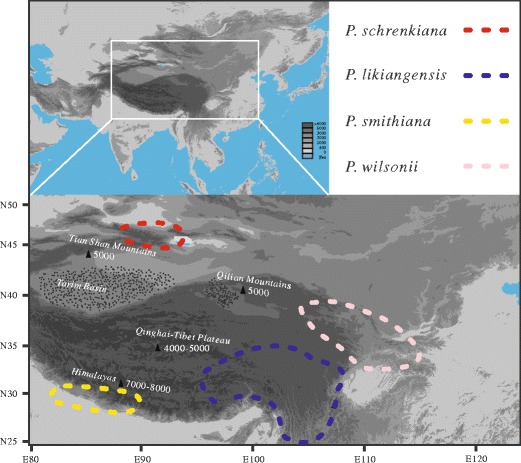
Distributions of *P. schrenkiana*, *P. smithiana*, *P. likiangensis*, and *P. wilsonii. P. schrenkiana* distributed in Tian Shan Mountains and neighboring areas (TSMs), *P. smithiana* distributed in Himalaya Mountains, *P. likiangensis* and *P. wilsonii* distributed in Qinghai–Tibet plateau and adjacent regions (QTPs). *Different colors* represent different species. The mean altitudes of mountains are given in *Arabic numerals*. Extensive geographical barriers, including deserts, are indicated by the areas of *black dots*; high mountains between the TSMs and the QTPs are shown by *small triangles*

Numerous alpine plant species are endemic in the Qinghai–Tibet Plateau (QTP), the Himalayas, and the Asian highlands—regions which together are recognized as one of the world’s most important alpine biodiversity hotspots (Wilson 1992; Myers et al. 2000). These endemic species may have originated during and/or after the extensive uplifts of the QTP and Himalayas. However, the date when both the QTP and Himalayas were uplifted to their current heights remains debatable, three extensive uplifts have been dated between 22 and 20, 15–8 and 5–3 Mya (e.g., Shi et al. 1998; An et al. 2001; Guo et al. 2002; Buslov et al. 2007; Wang et al. 2008). Previous studies have found that a few herbaceous genera diversified greatly during these three stages, especially the latter two (e.g., Liu et al. 2002, 2006; Wang et al. 2009). Studies designed to examine the origin and speciation of alpine trees, especially studies based on population genetic data, are rare (but see Mao et al. 2010 and Xu et al. 2010). In the present paper, we examine the phylogenetic relationships and speciation patterns of two Asian highland spruce species: *Picea schrenkiana* and *Picea smithiana*. The genus *Picea* diversified greatly in Asia with 24 out of the total of 35 spruce species occurring there (Fu et al. 1999; Farjón 2001). Most of them are distributed in cold and temperate mountainous regions, especially in the QTP and adjacent highlands. However, *P. schrenkiana* and *P. smithiana* occur with narrow distributions in the Central Asian Highlands (Tian Shan Mountains; Zhang and Tang 1989) and the Himalayas, respectively. These two species are isolated from each other by the western Kunlun Mountains, and both are isolated from spruces in the QTP by the Himalaya and Tarim depressions. All these geographic isolations seem to have developed before the Pliocene (5.3–2.5 Mya; Fig. 1). For example, the QTP–Himalayas uplift and Tarim depression occurred at the same as the first extensive uplift of the QTP around 22 Mya (Guo et al. 2002). The second extensive QTP uplift, which occurred between 15 and 8 Mya, was accompanied by further increases of the western Kunlun Mountains and the Himalayas, as well as the Tarim depressions (Wang et al. 1990, 2006, 2008; Abdrakhmatov et al. 1996; Shi et al. 1998; An et al. 2001; Bullen et al. 2001, 2003; Charreau et al. 2006, 2009; Buslov et al. 2007; Dupont–Nivet et al. 2008). It is likely that the origin and speciation of *P. schrenkiana* and *P. smithiana* are correlated with geographical isolations consequent upon these geological events.

These two species are morphologically distinct from each other, despite having similar quadrangular leaves: *P. schrenkiana* usually has thick leaves while leaves of *P. smithiana* are linear and more slender. These two species were placed together in the section *Picea* (Liu 1982; Farjón 2001) along with other species (e.g., *Picea wilsonii*, *Picea neoveitchii*, *Picea crassifolia*, and *Picea abies*) with quadrangular leaves and stomatal lines present and almost equal in number on each surface (Fu et al. 1999). Those species represented by *Picea likiangensis* of Sect. *Casicta* (Liu 1982; Farjón 2001) have flattened leaves with stomatal lines mostly or only present on the adaxial surface. However, phylogenetic analyses based on chloroplast (cp) DNA sequence variations suggest that both *P. schrenkiana* and *P. smithiana* cluster together with *P. likiangensis* into a well-supported subclade, sister to that represented by *P. wilsonii*. The remaining species of Sect. *Picea* form a separate clade with distant relationships (Ran et al. 2006; Bouillé et al. 2011). In contrast, phylogenetic analyses based mainly on variations in the mitochondrial (mt) genome suggest that *P. schrenkiana* clusters into a subclade with *P. wilsonii* while *P. smithiana* should be placed at the basal position of the subclade containing *P. likiangensis* (Lockwood et al. 2013). Another recent phylogenetic analysis based on 11 concatenated nuclear gene sequences suggested that *P. schrenkiana* and *P. smithiana* clustered into a separate clade, sister to the other one comprising the other eight species (Sun et al. 2014). It should be noted that all other species, i.e., *Picea spinulosa*, *Picea farreri*, *Picea neoveitchii*, *Picea morrisonicola*, *Picea purpurea*, and *Picea brachytyla* are closely related to *P. likiangensis* or *P. wilsonii*, or are derived from an original hybrid of these two key species (Liu 1982; Fu et al. 1999; Ran et al. 2006; Bouillé et al. 2011; Lockwood et al. 2013; Zou et al. 2013; Sun et al. 2014). We therefore chose *P. likiangensis* and *P. wilsonii* to represent the other species and further examined their phylogenetic relationships with *P. schrenkiana* and *P. smithiana* based on three genomes data. We sequenced three cpDNA loci, two mtDNA loci and 11 nuclear DNA loci from 30 populations comprising 330 individuals of four species. We used approximate Bayesian computation (ABC) methods (Wegmann et al. 2010) to test alternative speciation hypotheses between these four species, estimate the divergence timescales, and examine whether gene flow occurred during speciation events.

## Material and Methods

### Population Sampling

Populations of four species were sampled from across their major distributions and three varieties recognized under *P. likiangensis* were included (Fig. 1; Supplementary Table 1). The number of sampled individuals in each population was between 4 and 21, spaced ≥100 m apart. A total of 330 individuals were used in this study. Fresh leaf needles were dried and stored in the field using silica gel; seeds were stored at −20 °C. The latitude, longitude, and altitude of each sampling location were measured using an Extrex GIS monitor (Germany) (Supplementary Table 1). One *Picea meyeri* individual was used as an outgroup for phylogenetic analyses.

### Sequencing and Phasing

We used either the modified cetyltrimethylammonium bromide (CTAB) procedure (Doyle and Doyle 1990) or a QIAGEN DNeasy Plant Mini Kit (QIAGEN, Valencia, CA, USA) to extract the total genomic DNA from needles. Eleven nuclear loci (*4CL*, *EBS*, *GI*, *MOO2*, *M007D1*, *Sb16*, *Sb29*, *Sb62*, *se1364*, *se1390*, and *xy1420*) were selected and sequenced following Li et al. (2010) using an ABI 3130xl or 3730xl Genetic Analyzer (Applied Biosystems, Foster City, CA, USA) and an ABI Prism BigDye Terminator Cycle V3.1 Sequencing Kit. We amplified and sequenced three chloroplast loci (*trn*L–*trn*F, *trn*S–*trn*G, and *ndh*K/C; Taberlet et al. 1991; Hamilton 1999; Anderson et al. 2006) and two mitochondrial loci (*nad*1 intron b/c and *nad*5 intron1; Meng et al. 2007). Basecalling of nuclear genotypic sequences was done using Phred v0.020425.c (score >20) (Ewing et al. 1998) with CODONCODE ALIGNER software (CodonCode Corporation) and manually checked whether the SNPs consistent with the chromatogram peaks using the MEGA5 (Tamura et al. 2011). Sequences from cp and mt genomes were identified and checked using MEGA5. Finally, we used CLUSTAL W (Thompson et al. 1997) in MEGA5 to perform the alignment of sequence matrix at each locus.

Nuclear sequences with heterozygous sites were rephased and separated into two allele sequences by PHASE (Stephens et al. 2001, 2003) in the software package DnaSP V5 (Librado and Rozas 2009). A Hardy–Weinberg equilibrium was assumed and the phased sequences were inferred using a coalescent Bayesian method. Homozygous genotypes, and genotypes with single heterozygous sites, were set as known alleles to improve the performance of PHASE analyses. A general recombination model (Li and Stephens 2003) and 10 iterations of the final run were used and other optional parameters were set as default. Runs were repeated twice with different seed numbers to ensure that results were robust. In this research, three nuclear fragments (*Sb16*, *Sb29*, and *Sb62*,) were cloned and 10 clones were sequenced.

All newly obtained sequences of *P. schrenkiana* and *P. smithiana* were submitted to GenBank (accession numbers KJ176997–KJ179244) and three genomes sequences of *P. likiangensis* and *P. wilsonii* were cited from Li et al. (2013) and Zou et al. (2013).

### Phylogenetic Analyses

For cpDNA, mtDNA, and nuclear DNA, we used NETWORK V4.2.1.1 (Bandelt et al. 1999) (available at http://www.fluxus-engineering.com/) to construct their genealogies. We further used a Bayesian coalescent-based method for species tree estimation based on three genomes data, as implemented in BEAST 1.7.2 package (Drummond and Rambaut 2007; available at http://beast.bio.ed.ac.uk/Main_Page). The approach makes use of multilocus nuclear data, embedding individual gene trees in a multilocus species tree, allowing estimation of species genealogy (Hailer et al. 2012). Nonrecombining blocks of each locus were chosen using IMGC software (Woerner et al. 2007) because recombination cannot be modeled in the coalescence approach in BEAST. Using a strict molecular clock and the models of sequence evolution indicated by jModeltest (Posada 2008), we performed three independent runs of 2 × 10^8^ generations and sampled parameters every 5,000 iterations, with a burn-in of 30 %. Convergence was checked in Tracer 1.4 (available at http://beast.bio.ed.ac.uk/Tracer). For both cpDNA and mtDNA, all indels were coded as “0” and “1”. Branch supports were also evaluated using the program MrBayes 3.1.2 (Huelsenbeck et al. 2001; Ronquist and Huelsenbeck 2003).

### Population Genetic Parameters and Neutrality Tests

We used DnaSP V5 (Librado and Rozas 2009) to calculate basic population genetic parameters: the number of segregating sites (*S*), Watterson’s parameter (*θ*_*w*_, Watterson 1975), nucleotide diversity (*π*, Tajima 1983). For nuclear loci, we further showed the divergence between each pair of species using ARLEQUIN, version 3.0 (Excoffier et al. 2005) with significance tests based on 10,000 permutations.

Before estimating evolutionary history, we used a recently developed maximum frequency of derived mutation (MFDM) method (Li 2011) to test the neutrality of variation at each locus. The MFDM method is not affected by the impact of varying population size, such as expansion or shrinkage, which may produce similar signals to selection (Li 2011). Because the demographic dynamics cannot change the genealogies of a locus (Tajima 1983; Hein et al. 2004), an unbalanced topology shaped by recent selection could be captured and stand as evidence of non-neutrality. Simulation analysis and comparisons with several other methods showed higher power and a lower false-positive rate when detecting recent selection using the MFDM method (Li 2011). Here, we tested the neutrality per locus in each species with this method using a 5 % significance level. Ancestral states at variable sites were determined by comparison with two outgroup species (*Picea breweriana* and *P. meyeri*).

### Linkage Disequilibrium and Population Structure

Linkage disequilibrium (LD) was measured by *r* ^2^, the square of the correlation coefficient between each single nucleotide polymorphism (SNP) pair (Heuertz et al. 2006), using DnaSP V5 (Librado and Rozas 2009). The significance level of the statistical association between alleles at different sites was measured using Fisher’s exact test, and Bonferroni correction was used to correct for false positives. *r* ^2^ = 0 means loci are in complete linkage equilibrium and *r* ^2^ = 1 indicates loci are in complete linkage disequilibrium. However, the result showed that *r* ^2^ values were very small, almost equal to zero, which indicated loci were nearly no linkage disequilibrium (Supplementary Table 2). Therefore, we used all SNPs of nuclear loci to identify the genetic structure and inter relationships of four species at the population level by STRUCTURE V2.3.4 analysis (Pritchard et al. 2000; Hubisz et al. 2009). The most likely number of populations in the dataset (*K*) was estimated by conducting 15 independent runs for each *K* value ranging from 1 to 6. Each run had a burn-in of 200,000 iterations and additional 500,000 iterations. Finally, we used Distruct v.1.1 (Rosenberg 2004) to draw the graphics. We computed Δ*K*, using the method in Evanno et al. (2005), to determine the most likely number of clusters.

### Tests for Speciational Bottlenecks and Gene Flow Using ABCtoolbox

**Fig. 2:**
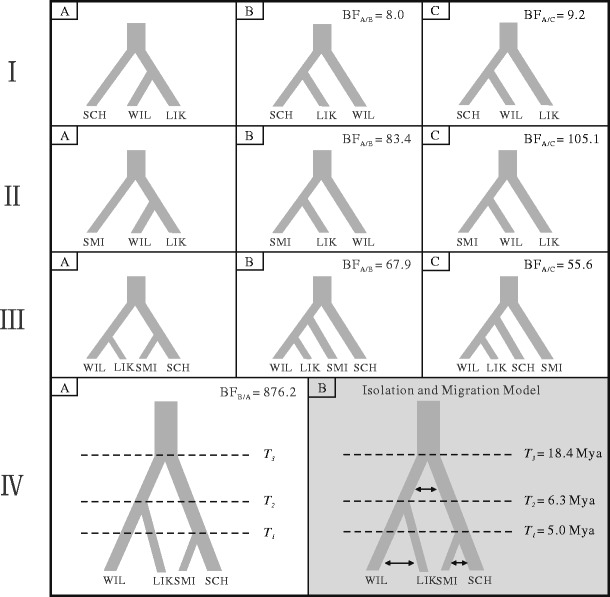
Models for origin of *P. schrenkiana* (SCH), *P. smithiana* (SMI), *P. likiangensis* (LIK) and *P. wilsonii* (WIL). If BF >3, model A was better than model B/C or model B was better than model A. *T1*, *T2*, and *T3* indicates the divergence time between *P. schrenkian* and *P. smithiana*, *P. likiangensis* and *P. wilsonii*, and *P. likiangensis*–*P. wilsonii* and *P. schrenkiana*–*P. smithiana*. The *black arrows* indicate the migrations between pairs of species

We compared four speciation models with and without gene flow using Approximate Bayesian Computation (ABC) implemented in the ABCtoolbox software package (Wegmann et al. 2010) based on the 11 nuclear loci sequence data. We then used Bayesian factors (BF) to choose the more suitable of two compared models, if BF >3, model A was better than model B or C (Fig. 2). We adopted four steps to test speciation orders between four species in order to reduce the number of the hypothesized models (together 64 models) and shorten modeling time (together more than 1 year). Firstly, we examined the interspecific relationships among *P. schrenkiana*, *P. likiangensis*, and *P. wilsonii* based on all three possible models. Secondly, we tested whether *P. smithiana* is closely related to *P. likiangensis* or *P. wilsonii* using a similar approach to the first step. Both tests from the first and second steps suggested that *P. likiangensis* and *P. wilsonii* always clustered together relative to *P. schrenkiana* or *P. smithiana* (Supplementary Table 3). Thirdly, we treated *P. likiangensis*–*P. wilsonii* as one clade, and examined its relationships with *P. schrenkiana* and *P. smithiana*. This test also suggested that *P. smithiana* and *P. schrenkiana* clustered together, diverging late from the *P. likiangensis*–*P. wilsonii* clade. Finally, we compared two different models with or without gene flow during these series of speciation events.

The current population sizes of *P. schrenkiana*, *P. smithiana*, *P. likiangensis*, *P. wilsonii*, and ancestor species were recorded as *NSCH*, *NSMI*, *NL*, *NW*, *N1* (ancestor of *P. likiangensis* and *P. wilsonii*), *N2* (ancestor of *P. schrenkiana* and *P. smithiana*), and *N3* (ancestor of all four species), respectively. The divergence time was recorded as *T1*, *T2*, and *T3*, representing the divergence time between *P. schrenkiana* and *P. smithiana*, *P. likiangensis* and *P. wilsonii*, and *P. likiangensis*–*P. wilsonii* and *P. schrenkiana*–*P. smithiana*, respectively. *Nm* was used to represent the migration numbers per generation.

We assumed uniform priors on the log_10_ scale for all population sizes (for *N1* (4.5, 5.7), for *N2* (3.8, 5.0), for *N3* (3.8, 6.0), for *NL* (5.3, 5.5), *NW* (5.4, 5.6), and for *NSCH*/*NSMI* (4.5, 5.0)) and three timing parameters (for *T1* (5.1, 5.3), for *T2* (5.2, 5.5), and for *T3* (5.5, 5.8)). We used 34 statistics computed by ARLEQUIN v3 (Excoffier et al. 2005) to summarize the population genetic information of *P. schrenkiana* or *P. smithiana* together with *P. likiangensis* and *P. wilsonii*, and we used 34 statistics to estimate the speciation models of four species. Thus, for each species, we computed the number of segregating sites, the number of private segregating sites, Tajima’s *D* (Tajima 1989), Fu and Li’s D* (Fu et al. 1999), and the number of pairwise differences. For each pair of species, we computed *F*_ST_, the average number of pairwise differences and the significance was tested by 1,000 permutations as implemented in ARLEQUIN version 3.0 (Excoffier et al. 2005). We also computed the total number of segregating sites over both species. To decrease the redundancy of statistics, we extracted partial least squares (PLS) components from the total of 34 summary statistics using the specific R script of ABCtoolbox (Wegmann et al. 2009). A total of 28 PLS components were used according to root mean square error plots and the conversion equations were inferred from the 10,000 samples simulated by a standard algorithm for each of the last speciation models. We used the likelihood-free ABC–MCMC (Markov chain Monte Carlo) methods (Wegmann et al. 2009) and the program fastsimcoal (Excoffier and Foll 2011) to simulate a total of 1,000,000 samples with a proposed range of *φ* = 1 and tolerance *δ* = 1 %. The 10,000 simulated samples used for inferring PLS components were used as the calibration in the MCMC sampling step in this study. We retained the 10,000 simulated samples that compared closest with the observed summary statistics of actual dataset following the PLS transformations and applied the regression adjustment general linear model (GLM) to generate posterior distributions of all parameters in each model. Parameters were estimated based on a mean mutation rate of *μ* = 1.41 × 10^−8^ per site per generation, with a conservative confidence interval between an extremely “slow” rate (1.11 × 10^−8^ per site per generation) and an extremely “fast” rate (1.71 × 10^−8^ per site per generation). Average generation time was set to 50 years according to previous studies for spruce species (Bousquet and Bouillé 2005; Chen et al. 2010) and the dated timescales need further confirmation from independent evidence. This generation time is about three to five times to the age at the first reproduction, but less than the maximum life expectancy of spruce species (Burns and Honkala 1990). It is likely that alpine spruces in our studied region may have longer generation times than those distributed in the low-altitude region. Even if these demographic parameters are underestimated, our population genetic estimates are of the same order of magnitude and largely consistent with those from other studies of spruce species (Bousquet and Bouillé 2005; Chen et al. 2010; Li et al. 2010). To detect gene flow after speciation, we further used IMa2 (Hey 2010a, 2010b) to estimate the interspecific gene flow after divergence based on the isolation-with-migration (IM) model (Nielsen and Wakeley 2001; Hey and Nielsen 2004, 2007; Hey 2010a, 2010b).

### Demographic Expansion Analysis

To assess the demographic history of four spruce species, we used LAMARC v2.1.8 (Kuhner 2006; available at http://evolution.genetics.washington.edu/lamarc/index.html), with a coalescent simulation approach that considered the genealogical relationships among the nuclear haplotypes to estimate the exponential population growth rate parameter (*g*) within the two species. The analysis was based on a Bayesian method, using Metropolis-coupled MCMC with replication of chains and adaptive heating to obtain better sampling of the parameter space. Large, positive values of the exponential growth parameter (*g*) indicate population expansion, but negative values indicate population shrinkage while relatively small positive values (*g* = 10) may indicate little or no growth.

## Results

### Variation in Chloroplast and Mitochondrial Loci and Interspecific Relationships

**Fig. 3:**
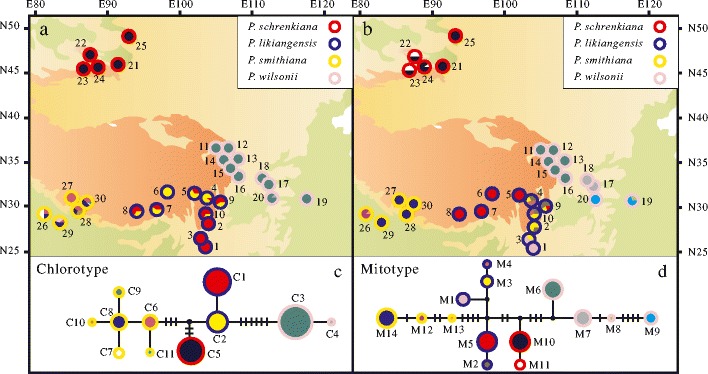
Distributions (**a**, **b**) and networks (**c**, **d**) of chlorotypes and mitotypes within four species: *P. schrenkiana*, *P. smithiana P. likiangensis*, and *P. wilsonii*. The *color of the circumference of the circles* indicates each species. The proportion of each *colored sector filling the circles* in **a** and **b** indicates the frequency of each chlorotype or mitotype in each species. Each haplotype in **c** and **d** is represented by a *circle* whose size is proportional to its frequency over all populations

**Fig. 4:**
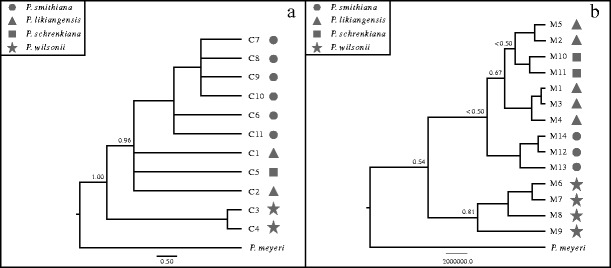
Phylogenetic trees constructed for chlorotypes (**a**) and mitotypes (**b**) recovered from all sampled individuals among four spruce species. *Numbers above nodes* indicate statistical significance

A total of 11 chlorotypes were identified based on the concatenated data of three chloroplast loci (*trn*L–*trn*F, *trn*S–*trn*G, and *ndh*K/C) across all individuals of the four spruce species (Supplementary Table 4). Chlorotypes of four species were grouped into two distinct groups and no haplotype was shared between species (Fig. 3). Two haplotypes from *P. wilsonii* comprised a single distinct clade with six steps detected. The other comprised six hapltoypes (C6–C11) from *P. smithiana*, a single haplotype C5 from *P. schrenkiana*, and two (C2 and C1) from *P. likiangensis*. C5 appeared to be more closely related to C2 and C1 than to C6–C11 in the network. Phylogenetic analyses also identified two distinct clades, and close relationships between haplotypes from *P. schrenkiana* and *P. likiangensis* were not supported by the statistics (Fig. 4).

Fourteen mitotypes were identified across all samples from the examination of the sequence variation based on concatenated data of two mitochondrial loci (*nad5* intron 1 and *nad1* intron b/c; Supplementary Table 5). No mitotype was shared between any two species. Similarly, two distinct groups were identified according to both network and phylogenetic analyses. Four mitotypes M6 to M9 from *P. wilsonii* (Fig. 3) comprised one clade at five steps from the other three species. In the other clade, two mitotypes (M10 and M11) in *P. schrenkiana* appeared to be closer to those (M1 to M5) found in *P. likiangensis* although such a relationship was not well supported (Fig. 4).

### Variation in Nuclear Loci and Interspecific Relationships

We sequenced 11 unlinked nuclear loci with a total concatenated length of 4,986 bp. We revealed 32, 38, 133, and 143 segregating sites in *P. schrenkiana*, *P. smithiana*, *P. likiangensis*, and *P. wilsonii*, respectively. *P. schrenkiana* and *P. smithiana* had a low level of silent nucleotide diversity, *π*_*s*_ (0.0012) and *π*_*s*_ (0.0016). A similar pattern was observed for the total nucleotide diversity (Table 1). *P. likiangensis* showed the highest nucleotide diversity in cytoplasmic DNA. MFDM tests revealed nearly no substantial evidence for selection acting on each locus across all four species (Table 2).

**Table 1:** Average nucleotide diversity across three different genomes in each of the four spruce species

Genomes	Species	Total					Nonsynonymous sites				Silent sites			
		*n*	*L*	*S*	*θ*_*W*_	*π*	*L*	*S*	*θ*_*W*_	*π*	*L*	*S*	*θ*_*W*_	*π*
nrDNAs	*P. schrenkiana*	60	4,986	32	0.0014	0.0012	2,444.37	14	0.0012	0.0012	2,532.63	18	0.0015	0.0012
	*P. smithiana*	98	4,986	38	0.0015	0.0017	2,442.88	17	0.0014	0.0018	2,534.12	20	0.0015	0.0016
	*P. likiangensis*	100	4,986	133	0.0053	0.0056	2,445.49	NA	NA	0.0042	2,531.51	NA	NA	0.00688
	*P. wilsonii*	72	4,986	143	0.006	0.0054	2,445.25	59	0.005	0.0044	2,531.75	86	0.007	0.0064
mtDNAs	*P. schrenkiana*	53	1,361	1	0.00016	0.00025	107.33	0	0	0	1,253.67	1	0.00018	0.00027
	*P. smithiana*	49	1,413	3	0.00048	0.00026	107.33	0	0	0	1,305.67	3	0.00052	0.00028
	*P. likiangensis*	91	1,367	5	0.00072	0.00101	107.33	0	0	0	1,259.67	5	0.00078	0.00109
	*P. wilsonii*	86	1,256	4	0.00063	0.00127	107.33	0	0	0	1,148.67	4	0.00069	0.00139
cpDNAs	*P. schrenkiana*	53	1,693	0	0	0	–	–	–	–	–	–	–	–
	*P. smithiana*	49	1,567	4	0.00057	0.00055	–	–	–	–	–	–	–	–
	*P. likiangensis*	91	1,699	9	0.00104	0.00039	–	–	–	–	–	–	–	–
	*P. wilsonii*	86	1,704	1	0.00012	0.00003	–	–	–	–	–	–	–	–

*n* sample size, *L* length in base pairs, *S* number of segregating sites, *θ*_*W*_ Watterson’s parameter, *π* nucleotide diversity, *NA* failed to compute

**Table 2:** *P* values of neutrality tests at each locus as measured by MFDM tests

Locus	*P. schrenkiana*	*P. smithiana*	*P. likiangensis*	*P. wilsonii*
*4CL*	NA	1.0000	0.6061	0.2254
*EBS*	1.0000	1.0000	1.0000	0.7887
*GI*	1.0000	1.0000	0.4444	1.0000
*MOO2*	1.0000	0.2062	0.0808	0.3380
*M007D1*	1.0000	1.0000	0.6869	1.0000
*Sb16*	1.0000	0.0512	0.7071	0.3099
*Sb29*	0.3389	1.0000	1.0000	1.0000
*Sb62*	0.3729	0.2062	0.7677	0.5634
*se1364*	1.0000	0.7835	NA	1.0000
*se1390*	0.2034	0.5361	0.6869	0.6761
*xy1420*	NA	NA	1.0000	1.0000
*nad1* intron b/c	1.0000	1.0000	1.0000	1.0000
*nad5* intron1	NA	NA	0.2222	0.4471
*trn*L–*trn*F	NA	0.04433*	1.0000	1.0000
*trn*S–*trn*G	NA	NA	0.0222*	NA
*ndh*K/C	NA	NA	0.0223	NA

*NA* failed to compute due to insufficient variation

*P < 0.05; **P < 0.01; and ***P < 0.001

Eight haplotype genealogies constructed by NETWORK V4.2.1.1 are shown in Supplementary Fig. 1, and the remaining three are not shown because of too many haplotypes. *P. schrenkiana* was highly differentiated from the other four species (*se1390*, *se1364*, *Sb29*, and *MOO2*), while *P. smithiana* could be distinguished from the other species at only three loci (*se1390*, *GI*, and *4CL*). At the remaining loci, interspecific differentiations were low and indistinct. However, AMOVAs conducted on all nuclear data showed that divergence between each pair of species was highly significant (*P* < 0.001, Table 3) and the highest genetic divergence existed between *P. schrenkiana* and *P. smithiana* (Table 3) which may be caused by large geographic distance.

**Table 3:** *Φ*_ST_ values over all loci among four spruce species

	*P. schrenkiana*	*P. smithiana*	*P. likiangensis*	*P. wilsonii*
*P. schrenkiana*	–			
*P. smithiana*	0.6312***	–		
*P. likiangensis*	0.5923***	0.54811***	–	
*P. wilsonii*	0.5835***	0.5231***	0.2585***	–

*P < 0.05; **P < 0.01; and ***P < 0.001

**Fig. 5:**
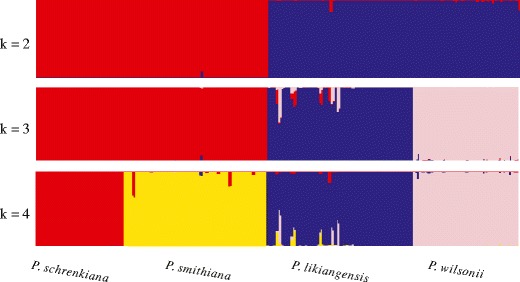
Structure analysis of four species when *K* = 2–4 clusters are assumed. For each *K* value, results of the run with the highest value of LnPD were used. Variation among runs was limited

**Fig. 6:**
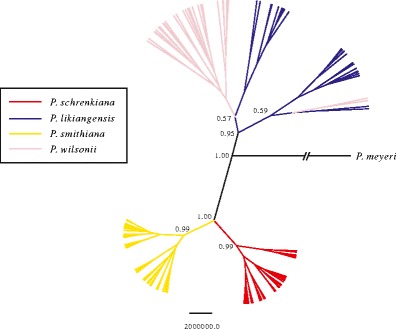
The phylogenetic tree based on the concatenated nuclear data using the BEAST 1.7.2. The *color of each line* indicates each species, and the *length of each line* shows the genetic distance

We then used a Bayesian clustering algorithm (Structure version 2.3.4) to construct the genetic structures of all examined populations and individuals. The Δ*K* (Evanno et al. 2005) tests showed that the most likely number of groups (*K*) for the entire dataset, using the total nuclear sequence data, was *K* = 4 and that the individuals of all four species clustered into four distinct clusters with high probability. In the clustering results for *K* = 2, the first cluster contained exclusively individuals from *P. schrenkiana* and *P. smithiana*, while the second cluster contained *P. likiangensis* and *P. wilsonii*. When *K* = 3, *P. schrenkiana* and *P. smithiana* still clustered into one group, but *P. likiangensis* and *P. wilsonii* clustered into two separate groups (Fig. 5, Supplementary Fig. 2). The phylogenetic tree based on the concatenated sequences of all nuclear loci indicated *P. schrenkiana* and *P. smithiana* clustered to one clade, and *P. likiangensis* and *P. wilsonii* formed the other with high-supporting values (Fig. 6). However, the individuals from *P. likiangensis* and *P. wilsonii* did not cluster into separate subclades, probably due to gene flow or intraspecific substructure.

**Table 4:** Maximum-likelihood estimates (MLE) and the 95 % highest posterior density (HPD) Intervals of migration rate among pair-wise comparisons

		MLE	HPD95Lo	HPD95Hi
*P. schrenkiana*/*P. smithiana*	*m*_1_	0.079	0	1.763
	*m*_2_	0.001	0	1.615
*P. schrenkiana*/*P. likiangensis*	*m*_1_	0.175	0	0.593
	*m*_2_	0.001	0	0.427
*P. schrenkiana*/*P. wilsonii*	*m*_1_	0.0645	0	0.3615
	*m*_2_	0.0015	0	0.6345
*P. smithiana*/*P. likiangensis*	*m*_1_	0.1375	0	0.8425
	*m*_2_	0.0025	0	1.127
*P. smithiana*/*P. wilsonii*	*m*_1_	0.1005	0	0.4995
	*m*_2_	0.0045	0	0.6885
*P. likiangensis*/*P. wilsonii*	*m*_1_	0.005	0	0.805
	*m*_2_	0.445	0.055	1.325

*m*_1_ is the migration rate from species 1 to species 2 forward in time, and *m*_2_ is the migration from species 2 to species 1

### Tests of Speciation Models and Demographic Histories of Four Species

The simulations with two demographic models of *P. schrenkiana*, *P. smithiana*, *P. likiangensis*, and *P. wilsonii* were obtained using approximate Bayesian computations (ABC–MCMC) methods and these simulated samples were used to calculate Bayes factors. All tests suggested the divergence between the four species to follow in the order: *P. schrenkiana*–*P. smithiana* versus *P. likiangensis*–*P. wilsonii*, then *P. likiangensis* versus *P. wilsonii*, and *P. schrenkiana* versus *P. smithiana*. In addition, the model that included gene flow between these speciation events (Model B; Fig. 2) provided a better fit to the observed nuclear data than did the model without gene flow (model B versus model A; BF = 876.2). ABC analyses (Fig. 2) further estimated that the divergence between *P. likiangensis*–*P. wilsonii* and *P. schrenkiana*–*P. smithiana* occurred around 18.4 Mya (95 % HPDI, 11.7–22.4 Mya) (Supplementary Table 6; Supplementary Fig. 3). The divergence between *P. schrenkiana* and *P. smithiana* was estimated at 5.0 Mya (95 % HPDI, 3.98–7.27 Mya) while *P. likiangensis* and *P. wilsonii* diverged at 6.31 Mya (95 % HPDI, 5.01–10.2 Mya). The results of ABC analyses revealed symmetric post-divergence gene flow between two ancestral clades and two pairs of the current species. We also examined gene flow between these four species using IMa2 program and our estimates (Table 4) were roughly consistent with the ABC results. In addition, large expansion population growth rate parameter (*g*) values of *P. schrenkiana* (378.99), *P. likiangensis* (1205.29), and *P. wilsonii* (35.92) showed all these species may have experienced sudden and recent expansions. The small (*g*) (0.0021) of *P. smithiana* indicated it to have had little or no growth.

## Discussion

In the present study, we combined population genetic analyses from chloroplast, mitochondrial, and nuclear genomes to examine the origin and speciation model of *P. schrenkiana* and *P. smithiana*, occurring in the Central Asian Highlands and the Himalayas. Although some remote populations were not sampled due to the difficulty to access, all studied populations can represent the core distributions of two species. The sampled individuals for some populations are fewer than expected because of the failure during gene amplifying and sequencing. Therefore, we focused our discussions and implications on the divergence and speciation between two species and their closely related congeners, rather than intraspecific divergences and diversity. In both chloroplast and mitochondrial loci, we found that *P. schrenkiana* is more closely related to *P. likiangensis* than to *P. wilsonii* or *P. smithiana*. However, nuclear datasets suggested that *P. schrenkiana* and *P. smithiana* are more closely related to each other than to either of the other two species. Modeling and testing of the speciation series of four species not only supported this conclusion but further suggested gene flow accompanying speciation. This inconsistency of phylogenetic relationships and speciation series of the four species between three genomes may derive from two different but interacting factors: (1) different rates of the incomplete lineage sorting; (2) interspecific gene flow accompanying speciation. Our further estimates of the speciation timescales between these four species suggest that their initial and further divergences seem to be correlated with the early QTP uplifts and the corresponding TSMs depressions.

### Incomplete Lineage Sorting and Interspecific Gene Flow

Three previous studies, each mainly based on chloroplast, mitochondrial, or nuclear sequence variations, but from fewer individuals, discovered a close affinity of *P. schrenkiana* with *P. likiangensis* (Ran et al. 2006) or *P. wilsonii* (Lockwood et al. 2013) or *P. smithiana* (Sun et al. 2014). In the present study, our phylogenetic analyses of both chloroplast and mitochondrial sequence variations suggest that *P. schrenkiana* seems to be more closely related to *P. likiangensis* (Fig. 4) than to *P. smithiana*. The clade containing haplotypes from these three species comprise a distinct clade that differs from that comprising the *P. wilsonii* haplotypes. However, both phylogenetic and population genetic analyses based on nuclear genetic sequence data suggest that *P. schrenkiana* and *P. smithiana* cluster first, while *P. likiangensis* and *P. wilsonii* together form the other clade (Fig. 6). It should be noted that within the latter clade, individuals from two species did not cluster into separate subclades. This would have resulted from substructures of each species and interspecific gene flow or introgressions, which may have distorted phylogenetic analyses of population data. In fact, STRUCTURE analysis clearly supported these two distinct species although gene flow and introgression between them were detected for a few individuals (Fig. 5). The divergence series between four species were further confirmed by modeling and testing of the genetic population data from the nuclear loci (Figs. 2 and 6). The inconsistent interspecific relationships at different genomes may have been caused by the following two factors. First, In contrast to the biparental inheritance exhibited by nuclear DNA (nrDNA), mtDNA is maternally transmitted through seeds, while cpDNA is paternally inherited via pollen in spruce species (Corriveau and Coleman 1988; Harris and Ingram 1991; Reboud and Zeyl 1994); therefore, the average coalescence time of two randomly picked chloroplast or mitochondrial alleles (*N*_e_ generation) is half of the nuclear DNA alleles (2*N*_e_ generation; Nei and Tajima 1981). Therefore, DNA variation would be lost more easily and species-specific mutations would be accumulated more quickly in cytoplasmic DNAs than in nuclear DNAs, especially in extremely small isolated populations. This may account for species-specific haplotypes in these two cytoplasmic DNAs. For *P. smithiana* which was isolated with other species by Himalayas, the small effective population size may have led to a high level of accumulation of species-specific mutations in the cytoplasmic DNA, which probably distorted the true interspecific relationships in the results of earlier studies (Ran et al. 2006; Bouillé et al. 2011; Zou et al. 2013). However, at the nuclear loci, incomplete lineage sorting still remains for most sampled loci. The additive mutations from multiple nuclear loci therefore recovered a different interspecific relationship from those based on mitochondrial and chloroplast sequence variations. Second, gene flow and interspecific introgressions, especially cytoplasmic haplotypes occur frequently in spruce (Li et al. 2010; Du et al. 2011; Zou et al. 2012). It is therefore likely that *P. schrenkiana* captured mitochondrial and chloroplast haplotypes from *P. likiangensis*. In addition, modeling of the nuclear population genetic data suggests that gene flow continued for a long time after the divergence. Gene flow at nuclear loci maybe occured more frequently between *P. schrenkiana* and *P. smithiana* than between *P. wilsonii* and/or *P. likiangensis* (Supplementary Table 6). Therefore, it is also likely the regional nuclear gene flow might have grouped *P. schrenkiana* and *P. smithiana* together. All these lines of evidence suggested that speciation histories of these species are more complex than expected and that both incomplete lineage sorting and gene flow might have together resulted in different interspecific relationships discovered between cytoplasmic and nuclear genomes.

### Geological Isolation and Speciation History

According to our parameter estimates based on 4 × 10^6^ simulated samples and the current data pattern, two clades *P. schrenkiana*–*P. smithiana* and *P. likiangensis*–*P. wilsonii* diverged around 18.4 Mya. The further divergences between *P. likiangensis* and *P. wilsonii* and between *P. schrenkiana* and *P. smithiana* were estimated to occur at 6.31 and 5.0 Mya, respectively. It is interesting to note that the divergence timescales estimated here agree well with the geological evidence that the QTP (and Himalayas) were uplifted extensively during these stages. For example, the first large-scale QTP–Himalayas uplifts were dated to occur at 22 Mya, although the uplift of the QTP and Himalayas began about 50 Mya, which caused some peaks that were high enough to trigger Asian desertification (Guo et al. 2002). Between 10 and 8 Mya, the second uplift might have occurred, which further enhanced the aridity of the Asian interior and the onset of the Asian monsoon (An et al. 2001). This uplift may have occurred as early as 15 Mya, and in particular may have caused further increases of the western Kunlun Mountains, the Himalayas, and the vast depressions of the Tarim (Wang et al. 1990; Abdrakhmatov et al. 1996; Shi et al. 1998; An et al. 2001; Bullen et al. 2001, 2003; Charreau et al. 2006, 2009; Wang et al. 2006; Buslov et al. 2007; Dupont–Nivet et al. 2008; Wang et al. 2008). It is also likely that the first extensive uplift promoted the initial divergence between two clades, *P. likiangensis*–*P. wilsonii* and *P. schrenkiana*–*P. smithiana*. It is interesting to find this divergence was accompanied with gene flow according to the selected fitter model. The second large-scale uplift might have terminated this gene flow and also initiated further divergences between *P. likiangensis* and *P. wilsonii* and between *P. schrenkiana* and *P. smithiana*. The TSMs deserts formed at this stage (Sun et al. 2009) and the further increase of the Himalayas should have restricted natural migration or dispersal between *P. schrenkiana* and *P. smithiana* (Fig. 1). Therefore, these two extensive uplifts of the QTP (−Himalayas) might have played an important role in the speciation histories of these four species.

In addition to previous studies, which have demonstrated that several herbal genera originated or diversified greatly during the QTP uplift stages (e.g., Liu et al. 2002, 2006; Wang et al. 2009), our population genetic analyses have indicated that trees with long generation times may also have speciated in response to these uplifts. Further analyses of more tree speciation events in the QTP and adjacent regions are needed to demonstrate this generality. Overall, these findings together highlight the complex speciation histories of the alpine plants, and the importance of the geological events in promoting plant diversification.

## Electronic Supplementary Material

Below is the link to the electronic supplementary material.

Supplementary Fig. 1 Haplotypes genealogies for eight nuclear loci. The *color of each sector of a circle* indicates the frequency of a haplotype recorded in each spruce species. The *size of a circle* is proportional to the frequency of the haplotype across the four species. *Each color* represents a different species. *Branch lengths longer than one mutation step* are marked on each branch (GIF 31 kb)

High resolution image (EPS 2510 kb)

Supplementary Fig. 2 **a** Δ*K* analysis across six independent structure runs (*K* = 1–6), each with 15 repeats, assuming admixture and correlated allele frequencies. **b** The mean of LnPD with 60 runs considered (GIF 9 kb)

High resolution image (EPS 784 kb)

Supplementary Fig. 3 Posterior distributions for demographic parameters of model B in Fig. 2 (GIF 43 kb)
(GIF 44 kb)
(GIF 13 kb)

High resolution image (EPS 3526 kb) High resolution image (EPS 3518 kb) High resolution image (EPS 1114 kb)

Supplementary Table 1 Sampling sites, sample size, and haplotype distribution for 30 populations of *P. schrenkiana*, *P. smithiana*, *P. likiangensi*, and *P. wilsonii* (DOCX 34 kb)

Supplementary Table 2 *r* ^2^ values at each nuclear locus as measured by DnaSP to analyze the linkage disequilibrium (DOCX 16 kb)

Supplementary Table 3 Bayesian factors obtained from approximate Bayesian computation (ABC), if BF >3, model A was better than model B/C or model B was better than model A (DOCX 15 kb)

Supplementary Table 4 Variable sites of the aligned sequences of three chloroplast DNA fragments in 11 haplotypes (DOCX 23 kb)

Supplementary Table 5 Variable sites of the aligned sequences of two mitochondrial DNA fragments in 14 haplotypes (DOCX 25 kb)

Supplementary Table 6 Posterior mode estimate and 95 % highest posterior density interval (HPDI) for demographic parameters in the origin speciation model B in group IV (Fig. 2). *T*, *N*, and *Nm* indicates the divergence time between species, the effective population size of species, and the migration rate from first species to second species, respectively. The abbreviations of species are in accordance with Fig. 2 (DOCX 20 kb)
