# Supplementary figures and images for: Origin and speciation of Picea schrenkiana and Piceasmithiana in the Center Asian Highlands and Himalayas
Source: Plant Mol Biol Report. 2014 Aug 17;33(3):661–72. doi: 10.1007/s11105-014-0774-5 (PMC4432025; doi:10.1007/s11105-014-0774-5)

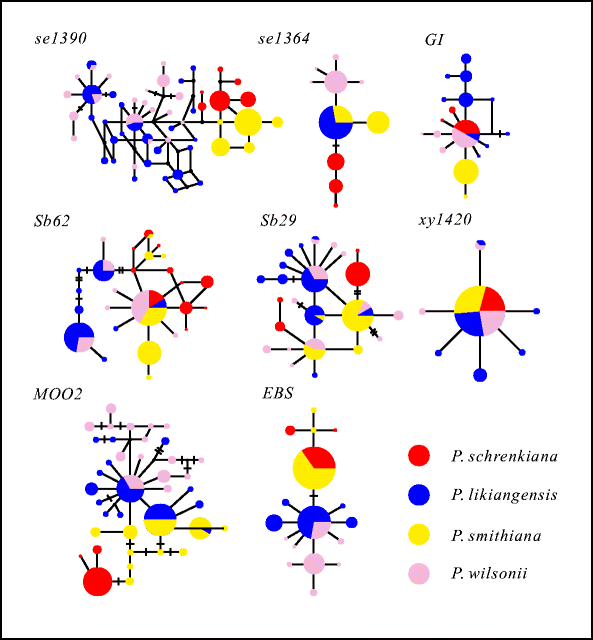

Supplement: Supplementary file 1 — Haplotypes genealogies for eight nuclear loci. The color of each sector of a circle indicates the frequency of a haplotype recorded in each spruce species. The size of a circle is proportional to the frequency of the haplotype across the four species. Each color represents a different species. Branch lengths longer than one mutation step are marked on each branch (GIF 31 kb) [file 11105_2014_774_Fig7_ESM.gif]

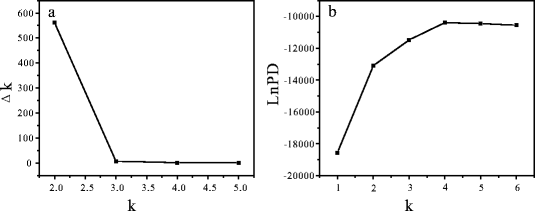

Supplement: Supplementary file 3 — a ΔK analysis across six independent structure runs (K = 1–6), each with 15 repeats, assuming admixture and correlated allele frequencies. b The mean of LnPD with 60 runs considered (GIF 9 kb) [file 11105_2014_774_Fig8_ESM.gif]

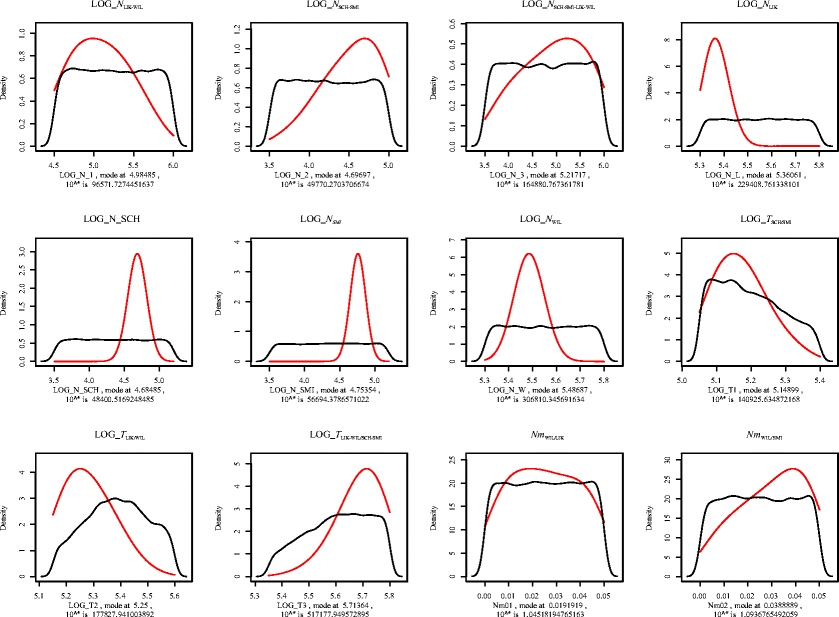

Supplement: Supplementary Fig. 3 — Posterior distributions for demographic parameters of model B in Fig. 2 (GIF 43 kb) (GIF 44 kb) (GIF 13 kb) [file 11105_2014_774_Fig9_ESM.gif]

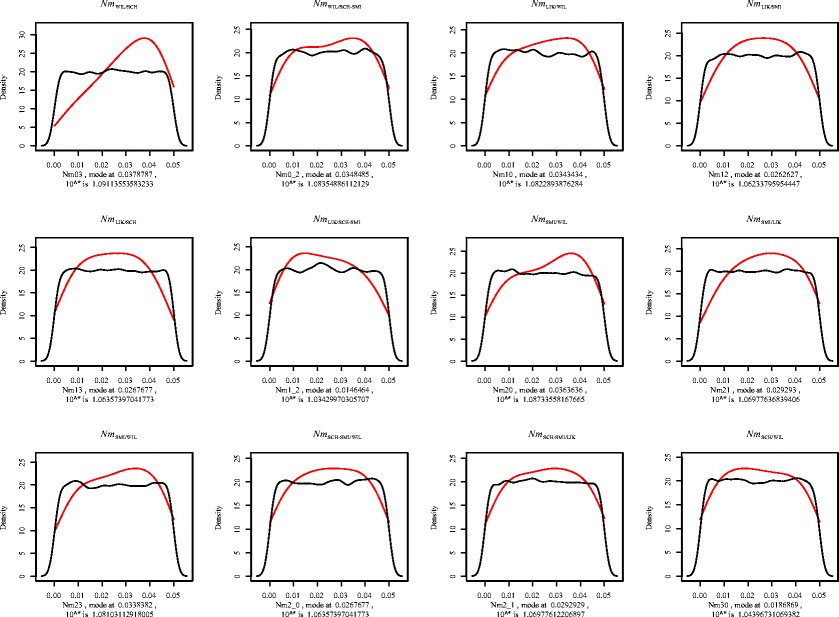

Supplement: Supplementary Fig. 3 — Posterior distributions for demographic parameters of model B in Fig. 2 (GIF 43 kb) (GIF 44 kb) (GIF 13 kb) [file 11105_2014_774_Fig10_ESM.gif]

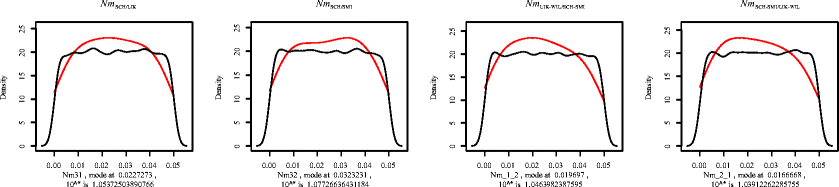

Supplement: Supplementary Fig. 3 — Posterior distributions for demographic parameters of model B in Fig. 2 (GIF 43 kb) (GIF 44 kb) (GIF 13 kb) [file 11105_2014_774_Fig11_ESM.gif]
