# Supplementary material for: Origin and speciation of Picea schrenkiana and Piceasmithiana in the Center Asian Highlands and Himalayas
Source: Plant Mol Biol Report. 2014 Aug 17;33(3):661–72. doi: 10.1007/s11105-014-0774-5 (PMC4432025; doi:10.1007/s11105-014-0774-5)
Supplement: Supplementary file 11 — Sampling sites, sample size, and haplotype distribution for 30 populations of P. schrenkiana, P. smithiana, P. likiangensi, and P. wilsonii (DOCX 34 kb) [file 11105_2014_774_MOESM6_ESM.docx]

**Supplementary Table 1** Sampling sites, sample size, and haplotype distribution for 30 populations of *P. likiangensi*, *P. wilsonii, P. schrenkiana* and *P. smithiana.*

| Code | Population | Long.(N) | Lat.(E) | Alt.(m) | Total | Chlorotype | | | | | | | | | | |  | Mitotype | | | | | | | | | | | | |  |
| --- | --- | --- | --- | --- | --- | --- | --- | --- | --- | --- | --- | --- | --- | --- | --- | --- | --- | --- | --- | --- | --- | --- | --- | --- | --- | --- | --- | --- | --- | --- | --- |
|  | |  |  |  |  | C1 | C2 | C3 | C4 | C5 | C6 | C7 | C8 | C9 | C10 | C11 |  | M1 | M2 | M3 | M4 | M5 | M6 | M7 | M8 | M9 | M10 | M11 | M12 | M13 | M14 |
| ***P. likiangensis*** | |  |  |  |  |  |  |  |  |  |  |  |  |  |  |  |  |  |  |  |  |  |  |  |  |  |  |  |  |  |  |
| 1 | Lijiang YN | 100 °14′ | 27°08′ | 3200 | 12 | 12 |  |  |  |  |  |  |  |  |  |  |  | 12 |  |  |  |  |  |  |  |  |  |  |  |  |  |
| 2 | Muli SC | 101°07′ | 27°58′ | 3550 | 10 | 12 |  |  |  |  |  |  |  |  |  |  |  |  | 3 | 7 |  |  |  |  |  |  |  |  |  |  |  |
| 3 | Diqin YN | 100°01′ | 27°36′ | 3000 | 13 | 13 |  |  |  |  |  |  |  |  |  |  |  | 2 |  | 1 |  |  |  |  |  |  |  |  |  |  |  |
| 4 | Daren SC | 100°54′ | 31°09′ | 3120 | 9 | 1 | 8 |  |  |  |  |  |  |  |  |  |  |  | 3 | 2 | 4 |  |  |  |  |  |  |  |  |  |  |
| 5 | Dege TB | 98°48′ | 31°55′ | 3741 | 9 | 3 | 6 |  |  |  |  |  |  |  |  |  |  |  |  |  |  | 9 |  |  |  |  |  |  |  |  |  |
| 6 | Leiwuqi TB | 96°25′ | 31°56′ | 4303 | 5 |  | 5 |  |  |  |  |  |  |  |  |  |  |  |  |  |  | 5 |  |  |  |  |  |  |  |  |  |
| 7 | Galongla TB | 95°42′ | 29°47′ | 3610 | 12 | 5 | 7 |  |  |  |  |  |  |  |  |  |  |  |  |  |  | 12 |  |  |  |  |  |  |  |  |  |
| 8 | Milin TB | 93°58′ | 29°12′ | 3000 | 9 | 6 | 3 |  |  |  |  |  |  |  |  |  |  |  |  |  |  | 9 |  |  |  |  |  |  |  |  |  |
| 9 | Kangding SC | 101°30′ | 30°15′ | 3100 | 5 | 3 | 2 |  |  |  |  |  |  |  |  |  |  |  | 3 | 1 |  | 1 |  |  |  |  |  |  |  |  |  |
| 10 | Litang YN (SC) | 100°19′ | 29°36′ | 4180 | 7 | 5 | 2 |  |  |  |  |  |  |  |  |  |  |  | 2 | 3 | 2 |  |  |  |  |  |  |  |  |  |  |
| ***P. wilsonii*** | |  |  |  |  |  |  |  |  |  |  |  |  |  |  |  |  |  |  |  |  |  |  |  |  |  |  |  |  |  |  |
| 11 | Huzhu QH | 102°28′ | 36°55′ | 2322 | 5 |  |  | 5 |  |  |  |  |  |  |  |  |  |  |  |  |  |  | 5 |  |  |  |  |  |  |  |  |
| 12 | Tulugou GS | 102°46′ | 36°40′ | 2400 | 5 |  |  | 5 |  |  |  |  |  |  |  |  |  |  |  |  |  |  | 5 |  |  |  |  |  |  |  |  |
| 13 | Xinglongshan GS | 104°03′ | 35°47′ | 2400 | 11 |  |  | 11 |  |  |  |  |  |  |  |  |  |  |  |  |  |  | 11 |  |  |  |  |  |  |  |  |
| 14 | Mengda QH | 102°40′ | 35°48′ | 2526 | 12 |  |  | 12 |  |  |  |  |  |  |  |  |  |  |  |  |  |  | 12 |  |  |  |  |  |  |  |  |
| 15 | Diebu GS | 103°12′ | 34°03′ | 3431 | 6 |  |  | 6 |  |  |  |  |  |  |  |  |  |  |  |  |  |  | 6 |  |  |  |  |  |  |  |  |
| 16 | Zhouqu GS | 104°21′ | 33°35′ | 1645 | 4 |  |  | 4 |  |  |  |  |  |  |  |  |  |  |  |  |  |  | 4 |  |  |  |  |  |  |  |  |
| 17 | Ningshan SN | 108°19′ | 33°18′ | 1198 | 3 |  |  | 3 |  |  |  |  |  |  |  |  |  |  |  |  |  |  |  | 3 |  |  |  |  |  |  |  |
| 18 | Foping SN | 107°49′ | 33°37′ | 903 | 21 |  |  | 20 | 1 |  |  |  |  |  |  |  |  |  |  |  |  |  |  | 21 |  |  |  |  |  |  |  |
| 19 | Shennongjia HU | 114°18′ | 30°29′ | 1829 | 10 |  |  | 10 |  |  |  |  |  |  |  |  |  |  |  |  |  |  |  |  | 3 | 7 |  |  |  |  |  |
| 20 | Chengkou CQ | 108°47′ | 31°58′ | 1353 | 9 |  |  | 8 | 1 |  |  |  |  |  |  |  |  |  |  |  |  |  |  |  |  | 9 |  |  |  |  |  |
| ***P. schrenkiana*** | |  |  |  |  |  |  |  |  |  |  |  |  |  |  |  |  |  |  |  |  |  |  |  |  |  |  |  |  |  |  |
| 21 | Wulumuqi XJ | 87°07′ | 43°12′ | 2147 | 12 |  |  |  |  | 12 |  |  |  |  |  |  |  |  |  |  |  |  |  |  |  |  | 12 |  |  |  |  |
| 22 | Yili XJ | 81°49′ | 43°20′ | 1967 | 10 |  |  |  |  | 10 |  |  |  |  |  |  |  |  |  |  |  |  |  |  |  |  | 5 | 5 |  |  |  |
| 23 | Zhaosu XJ | 81°07′ | 43°14′ | 2166 | 10 |  |  |  |  | 10 |  |  |  |  |  |  |  |  |  |  |  |  |  |  |  |  | 5 | 5 |  |  |  |
| 24 | Gongliu XJ | 82°31′ | 43°06′ | 1464 | 11 |  |  |  |  | 11 |  |  |  |  |  |  |  |  |  |  |  |  |  |  |  |  | 10 | 1 |  |  |  |
| 25 | Qitai XJ | 89°35′ | 49°36′ | 876 | 10 |  |  |  |  | 10 |  |  |  |  |  |  |  |  |  |  |  |  |  |  |  |  | 10 |  |  |  |  |
| ***P. smithiana*** | |  |  |  |  |  |  |  |  |  |  |  |  |  |  |  |  |  |  |  |  |  |  |  |  |  |  |  |  |  |  |
| 26 | Nepal | 81°08' | 29°25' | 2200 | 8 |  |  |  |  |  | 2 | 4 | 2 |  |  |  |  |  |  |  |  |  |  |  |  |  |  |  | 7 | 1 |  |
| 27 | Jilong | 85°15' | 28°50' | 2600 | 4 |  |  |  |  |  | 4 |  |  |  |  |  |  |  |  |  |  |  |  |  |  |  |  |  |  |  | 4 |
| 28 | Jilong | 85°19' | 28°22' | 2800 | 10 |  |  |  |  |  | 4 |  | 3 | 3 |  |  |  |  |  |  |  |  |  |  |  |  |  |  |  |  | 10 |
| 29 | Nepal | 84°50' | 28°31' | 2110 | 11 |  |  |  |  |  | 1 |  | 6 |  | 4 |  |  |  |  |  |  |  |  |  |  |  |  |  |  |  | 11 |
| 30 | Jilong | 85°20ʹ | 28°23ʹ | 2720 | 16 |  |  |  |  |  | 6 |  | 8 |  | 1 | 1 |  |  |  |  |  |  |  |  |  |  |  |  |  |  | 16 |

Notes: YN, Yunnan; SC, Sichuan; TB, Tibet; QH, Qinghai; SN, Shanxi; HU, Hubei; CQ, Chongqing; XJ, Xinjiang.
