# Supplementary material for: Origin and speciation of Picea schrenkiana and Piceasmithiana in the Center Asian Highlands and Himalayas
Source: Plant Mol Biol Report. 2014 Aug 17;33(3):661–72. doi: 10.1007/s11105-014-0774-5 (PMC4432025; doi:10.1007/s11105-014-0774-5)
Supplement: Supplementary file 12 — r 2 values at each nuclear locus as measured by DnaSP to analyze the linkage disequilibrium (DOCX 16 kb) [file 11105_2014_774_MOESM7_ESM.docx]

**Supplementary Table 2** r^2^ values at each nuclear locus as measured by DnaSP to analyze the linkage disequilibrium.

| *Locus* | *P. schrenkiana* | *P. smithiana* | *P. likiangensis* | *P. wilsonii* |
| --- | --- | --- | --- | --- |
| *4CL* | 0.0000 | 0.0000 | 0.0795 | 0.1516 |
| *EBS* | 0.0000 | 0.1400 | 0.0094 | 0.0090 |
| *GI* | 0.0036 | 0.0000 | 0.0963 | 0.0419 |
| *MOO2* | 0.0015 | 0.2104 | 0.0638 | 0.1040 |
| *M007D1* | 0.0009 | 0.0182 | 0.0544 | 0.0839 |
| *Sb16* | 0.0009 | 0.0655 | 0.0491 | 0.0805 |
| *Sb29* | 0.5180 | 0.5550 | 0.1017 | 0.1042 |
| *Sb62* | 0.1020 | 0.1489 | 0.3549 | 0.3190 |
| *se1364* | 0.0515 | 0.0000 | 0.0000 | 0.0007 |
| *se1390* | 0.2070 | 0.2197 | 0.1059 | 0.0735 |
| *xy1420* | 0.0000 | 0.0000 | 0.0012 | 0.0017 |
