# Supplementary material for: Origin and speciation of Picea schrenkiana and Piceasmithiana in the Center Asian Highlands and Himalayas
Source: Plant Mol Biol Report. 2014 Aug 17;33(3):661–72. doi: 10.1007/s11105-014-0774-5 (PMC4432025; doi:10.1007/s11105-014-0774-5)
Supplement: Supplementary file 13 — Bayesian factors obtained from approximate Bayesian computation (ABC), if BF >3, model A was better than model B/C or model B was better than model A (DOCX 15 kb) [file 11105_2014_774_MOESM8_ESM.docx]

**Supplementary Table 3** Bayesian factors obtained from Approximate Bayesian Computation (ABC), if BF>3, model A was better than model B/C or model B was better than model A.

| Steps | BF_A/B_ | BF_A/C_ |
| --- | --- | --- |
| I | 8.0 | 9.2 |
| II | 83.4 | 105.1 |
| III | 67.9 | 55.6 |
| IV | 876.2 |  |
