# Supplementary material for: Origin and speciation of Picea schrenkiana and Piceasmithiana in the Center Asian Highlands and Himalayas
Source: Plant Mol Biol Report. 2014 Aug 17;33(3):661–72. doi: 10.1007/s11105-014-0774-5 (PMC4432025; doi:10.1007/s11105-014-0774-5)
Supplement: Supplementary file 14 — Variable sites of the aligned sequences of three chloroplast DNA fragments in 11 haplotypes (DOCX 23 kb) [file 11105_2014_774_MOESM9_ESM.docx]

**Supplementary Table 4** Variable sites of the aligned sequences of three chloroplast DNA fragments in eleven haplotypes.

| Chlorotype | *trn*S*-trn*G | | | | | |  | *trn*L*-trn*F | | | | | | | |  | *ndh*K/C | | | |
| --- | --- | --- | --- | --- | --- | --- | --- | --- | --- | --- | --- | --- | --- | --- | --- | --- | --- | --- | --- | --- |
|  |  |  |  |  |  |  |  |  |  |  | 1 | 1 | 1 | 1 | 1 |  | 1 | 1 | 1 | 1 |
|  |  |  |  | 1 | 3 | 5 |  | 7 | 7 | 9 | 0 | 0 | 1 | 1 | 2 |  | 3 | 4 | 4 | 5 |
|  | 9 | 9 | 9 | 0 | 6 | 5 |  | 8 | 9 | 3 | 2 | 9 | 1 | 1 | 5 |  | 2 | 4 | 6 | 3 |
|  | 4 | 8 | 9 | 8 | 7 | 9 |  | 4 | 5 | 0 | 5 | 9 | 4 | 5 | 1 |  | 8 | 3 | 1 | 8 |
| C1 | C | C | C | ▼ | A | G |  | ▲ | T | C | A | — | G | A | C |  | A | A | C | ■ |
| C2 | C | C | C | ▼ | A | G |  | ▲ | T | C | A | — | G | A | C |  | C | A | C | ■ |
| C3 | C | C | C | ▼ | C | A |  | ▲ | T | C | C | ► | G | A | C |  | C | G | G | ■ |
| C4 | C | C | C | ▼ | C | A |  | ▲ | T | T | C | ► | G | A | C |  | C | G | G | ■ |
| C5 | T | C | C | ▼ | C | G |  | — | T | C | A | — | G | A | C |  | C | A | C | ■ |
| C6 | T | T | T | — | A | G |  | ▲ | T | C | A | — | G | A | C |  | C | A | C | ■ |
| C7 | T | T | T | — | A | G |  | ▲ | A | C | A | — | T | A | C |  | C | A | C | ■ |
| C8 | T | T | T | — | A | G |  | ▲ | T | C | A | — | T | A | C |  | C | A | C | ■ |
| C9 | T | T | T | — | A | G |  | ▲ | T | C | A | — | T | A | C |  | C | A | C | — |
| C10 | T | T | T | — | A | G |  | ▲ | T | C | A | — | T | C | C |  | C | A | C | ■ |
| C11 | T | T | T | — | A | G |  | ▲ | T | C | A | — | G | A | T |  | C | A | C | ■ |

Notes: — indicates missing nucleotides.

▼ CTGGC;

▲ TAAGAT;

► GATTT;

■ACAGTCTCCCTTTATCTATGGGCTATTAGTTCTAGTATACAGGGTATATCTGTATTTATAAGAGCTTCAATTCTCGTGCTTATTTTCATCGTTGGTTCTGTG CGAAAAGGAACATTGGAATGTTTC.
