# Supplementary material for: Origin and speciation of Picea schrenkiana and Piceasmithiana in the Center Asian Highlands and Himalayas
Source: Plant Mol Biol Report. 2014 Aug 17;33(3):661–72. doi: 10.1007/s11105-014-0774-5 (PMC4432025; doi:10.1007/s11105-014-0774-5)
Supplement: Supplementary file 15 — Variable sites of the aligned sequences of two mitochondrial DNA fragments in 14 haplotypes (DOCX 25 kb) [file 11105_2014_774_MOESM10_ESM.docx]

**Supplementary Table 5** Variable sites of the aligned sequences of two mitochondrial DNA fragments in fourteen haplotypes.

| Mitotype | *nad5* | | | | | | |  | *nad1* | | | | | | | | | | | | |  | | | |
| --- | --- | --- | --- | --- | --- | --- | --- | --- | --- | --- | --- | --- | --- | --- | --- | --- | --- | --- | --- | --- | --- | --- | --- | --- | --- |
|  |  |  |  | 1 | 3 | 3 | 6 |  | 6 | 6 | 6 | 6 | 6 | 6 | 6 | 6 | 6 | 6 | 6 | 6 | 6 | 7 | 9 | 9 | 9 |
|  | 9 | 9 | 9 | 5 | 0 | 0 | 7 |  | 7 | 7 | 7 | 7 | 7 | 7 | 7 | 8 | 8 | 8 | 8 | 8 | 9 | 0 | 7 | 7 | 8 |
|  | 3 | 5 | 6 | 6 | 0 | 6 | 1 |  | 3 | 4 | 5 | 6 | 7 | 8 | 9 | 0 | 1 | 2 | 3 | 4 | 9 | 8 | 2 | 3 | 0 |
| M1 | C | — | ▼ | — | T | C | — |  | ▀ | A | — | C | ♠ | A | ● | — | — | G | G | C | — | C | ☆ | — | T |
| M2 | A | — | — | — | T | C | — |  | ▀ | A | — | C | ♠ | C | ● | — | — | G | T | C | — | C | ☆ | — | T |
| M3 | A | — | — | — | T | C | — |  | ▀ | A | — | C | ♠ | A | ● | — | — | G | G | C | — | C | ☆ | — | T |
| M4 | A | — | — | — | T | C | — |  | ▀ | A | — | C | ♠ | A | — | — | — | G | G | C | — | C | ☆ | — | T |
| M5 | A | — | — | — | T | C | — |  | ▀ | A | — | C | ♠ | C | ● | — | — | G | G | C | — | C | ☆ | — | T |
| M6 | A | — | ▼ | — | G | C | — |  | — | C | — | C | ♠ | C | ● | ♥ | — | T | G | A | ◇ | C | — | — | T |
| M7 | A | — | ▼ | — | G | C | — |  | — | A | — | C | ♠ | C | ● | ♥ | — | T | G | A | ◇ | C | — | — | G |
| M8 | A | — | — | — | G | A | — |  | — | A | — | C | ♠ | C | ● | ♥ | — | T | G | A | ◇ | C | — | — | G |
| M9 | A | — | — | — | G | A | — |  | ▀ | A | — | C | — | C | ● | ♥ | — | T | G | C | — | C | — | — | T |
| M10 | A | — | ▼ | — | T | C | — |  | ▀ | A | — | C | ♠ | C | ● | ♥ | — | G | G | C | ◇ | A | — | — | T |
| M11 | A | — | ▼ | — | T | C | — |  | ▀ | A | — | A | ♠ | C | ● | ♥ | — | G | G | C | ◇ | A | — | — | T |
| M12 | A | ▲ | ▼ | ► | T | A | ◄ |  | ▀ | C | █ | C | ♠ | C | ● | — | ♀ | G | G | C | — | C | ☆ | — | G |
| M13 | A | ▲ | ▼ | ► | T | A | ◄ |  | ▀ | A | █ | C | ♠ | C | ● | — | — | G | G | C | — | C | ☆ | — | T |
| M14 | A | ▲ | ▼ | ► | T | A | ◄ |  | ▀ | C | █ | C | ♠ | C | ● | — | ♀ | G | T | C | — | C | ☆ | ○ | G |

Notes: — indicates missing nucleotides.

▲ AGGCT;

▼ TTAAT;

► CTTTACTTGAGACTTG;

◄ACGCCAATGTCG;

▀ TGTCTAAAG;

█TCTGGTGGGG;

♠ CAAGCCCTGTATTTATTCCCCTAAAAAAAAAAGTCTGGGCCGCCATAAATTAAAAGGGGCCATTAAAAGACTTTCCAGGGCCATGA;

● CATAAAG;

♥ AAATTA;

♀ GCCAT;

◇ TGTCT;

☆ GGAACGTAGTCGCTCGACCATAAGGGAGA;

○ TGTTAACGCTCACTCATCATATGGGTGAGGG.
