# Supplementary material for: Origin and speciation of Picea schrenkiana and Piceasmithiana in the Center Asian Highlands and Himalayas
Source: Plant Mol Biol Report. 2014 Aug 17;33(3):661–72. doi: 10.1007/s11105-014-0774-5 (PMC4432025; doi:10.1007/s11105-014-0774-5)
Supplement: Supplementary file 16 — Posterior mode estimate and 95 % highest posterior density interval (HPDI) for demographic parameters in the origin speciation model B in group IV (Fig. 2). T, N, and Nm indicates the divergence time between species, the effective population size of species, and the migration rate from first species to second species, respectively. The abbreviations of species are in accordance with Fig. 2 (DOCX 20 kb) [file 11105_2014_774_MOESM11_ESM.docx]

**Supplementary Table6**Posterior mode estimate and 95% highest Posterior Density Interval (HPDI) for demographic parameters in the origin speciation model B in group IV (Fig. 2).*T*, *N* and *Nm* indicates the divergence time between species, the effective population size of species and the migration rate from first species to second species, respectively. The abbreviations of species are in accordance with Fig. 2.

|  | Parameter | Model | Lower_bound | Upper_bound |
| --- | --- | --- | --- | --- |
| Divergence Time (Mya) | |  |  |  |
|  | *T*_SCH/SMI_ | 5.00 | 3.98 | 7.27 |
|  | *T*_LIK/WIL_ | 6.31 | 5.01 | 10.20 |
|  | *T*_LIK-WIL/SCH-SMI_ | 18.40 | 11.70 | 22.40 |
| Effective Population Size (×10^4^ individuals) | | | | |
|  | *N_SCH_* | 3.43 | 1.90 | 5.97 |
|  | *N_SMI_* | 4.02 | 2.50 | 6.46 |
|  | *N*_LIK_ | 16.30 | 14.20 | 20.30 |
|  | *N*_WIL_ | 21.80 | 16.50 | 28.80 |
|  | *N*_SCH-SMI_ | 3.53 | 0.48 | 7.09 |
|  | *N*_LIK-WIL_ | 6.85 | 2.24 | 37.80 |
|  | *N*_SCH-SMI-LIK-WIL_ | 11.7 | 0.43 | 70.90 |
| Migration Rate (×10^-2^ individuals / generation) | | | | |
|  | *Nm*_WIL/LIK_ | 1.36 | 0.18 | 3.40 |
|  | *Nm*_WIL/SMI_ | 2.76 | 0.47 | 3.54 |
|  | *Nm*_WIL/SCH_ | 2.69 | 0.47 | 3.54 |
|  | *Nm*_WIL/SCH-SMI_ | 2.47 | 0.18 | 3.43 |
|  | *Nm*_LIK/WIL_ | 2.44 | 0.22 | 3.47 |
|  | *Nm*_LIK/SMI_ | 1.86 | 0.23 | 3.39 |
|  | *Nm*_LIK/SCH_ | 1.90 | 0.15 | 3.32 |
|  | *Nm*_LIK/SCH-SMI_ | 1.04 | 0.08 | 3.29 |
|  | *Nm*_SMI/WIL_ | 2.58 | 0.30 | 3.46 |
|  | *Nm*_SMI/LIK_ | 2.08 | 0.29 | 3.51 |
|  | *Nm*_SMI/SCH_ | 2.40 | 0.30 | 3.46 |
|  | *Nm*_SCH-SMI/WIL_ | 1.90 | 0.15 | 3.40 |
|  | *Nm*_SCH-SMI/LIK_ | 2.08 | 0.18 | 3.43 |
|  | *Nm*_SCH/WIL_ | 1.33 | 0.15 | 3.36 |
|  | *Nm*_SCH/LIK_ | 1.61 | 0.15 | 3.32 |
|  | *Nm*_SCH/SMI_ | 2.29 | 0.15 | 3.36 |
|  | *Nm*_LIK-WIL/SCH-SMI_ | 1.40 | 0.08 | 3.25 |
|  | *Nm*_SCH-SMI/LIK-WIL_ | 1.18 | 0.08 | 3.29 |
